# Sensing Advancement and Health Monitoring of Transport Structures

**DOI:** 10.3390/s21227621

**Published:** 2021-11-16

**Authors:** Andrea Benedetto, Imad L. Al-Qadi, Amir M. Alani, Andreas Loizos, Fabio Tosti

**Affiliations:** 1 Department of Engineering, Roma Tre University, Via Vito Volterra 62, 00146 Rome, Italy; andrea.benedetto@uniroma3.it; 2 Department of Civil and Environmental Engineering, Illinois Center for Transportation, University of Illinois at Urbana-Champaign, Urbana, IL 61801, USA; alqadi@illinois.edu; 3 School of Computing and Engineering, University of West London (UWL), St Mary’s Road, Ealing, London W5 5RF, UK; Amir.Alani@uwl.ac.uk; 4 Laboratory of Pavement Engineering, Zografou Campus, National Technical University of Athens (NTUA), 9 Iroon Polytechniou Str., Zografou, 15780 Athens, Greece; aloizos@central.ntua.gr

Planning, design, construction, maintenance and management of transport infrastructure demand new methods and approaches to optimise utilisation of materials, energy and workforce. Assessing the performance of the infrastructure through novel monitoring during construction and throughout its life cycle must be a key target. Progress in sensing systems can be a steering factor to revolutionise outdated methods of infrastructure condition assessment and monitoring. Although most of our transport infrastructure is realised to perform over a long-lasting design life, its usage is driven by choices made by individuals and societal needs, which can apply to relatively variable time terms. These could affect the original design prediction significantly and lead to over or under design, including issues such as increased usage and infrastructure ageing. To tackle this problem, it is essential to provide active monitoring of construction and operational processes of transport infrastructure. This implies the provision of new protocols for remote and on-site routine monitoring of materials and structures and the assessment of their performance versus design factors or predictive models.

Monitoring in transport infrastructure engineering is being performed for decades, beginning with the use of visual inspections, where feedback was used to drive maintenance and upgrade design and construction practice. However, it is observed that the civil engineering sector has been often reluctant to adopt new sensing systems. This is due to a variety of factors, including relying on consolidated practice, budget constraints for research and innovation, challenges in matching industry and research time and needs, etc. Sometimes, existing technologies developed in other industrial sectors are adapted for use in transport infrastructure monitoring, affecting quality of results and costs of information.

More recently, research in sensing techniques has progressed tremendously. This is also driven by a change of perspective in managing physical infrastructure, i.e., from a cost-minimisation only to a value-based perspective, with potential to support asset owners in achieving the best value for money and safety levels during the infrastructure life cycle. There exist many sensor systems available for monitoring a variety of infrastructure attributes [1]. These include active and passive sensors, analogue and digital sensors, ground-based, embedded and remote sensing systems. Furthermore, the development of new smart infrastructure monitoring systems is now creating favourable conditions for use of sensors in both stand-alone and integrated multi-source operating modes [2]. Sensors based on acoustic, electrical, electromagnetic, chemical, optical and radioactive principles can be used in combination and the information merged to reach more effective infrastructure condition assessment and monitoring.

This Special Issue of Sensors aims to report significant recent advances in sensing systems for assessment and health monitoring of transport infrastructure and construction materials. The thirteen accepted papers cover theoretical, numerical and experimental developments across the whole range of transportation modes (highways, railways and airfields), including research findings in sensing techniques for construction materials.

Shokravi et al. [3] presents a review on vehicle-assisted techniques for structural health monitoring of bridges. A comprehensive overview is provided on methods such as vision-based, weigh-in-motion (WIM), bridge weigh-in-motion (BWIM), drive-by and vehicle–bridge interaction (VBI)-based models. Furthermore, the performance of vehicle-assisted methods is analyzed and future directions for research in this area are outlined. These include to compensate the drawbacks of individual approaches by using smart and autonomous vehicle-assisted monitoring methods.

With the aim to investigate into the mechanisms leading to shear failure on composite rocks, Liu et al. [4] report a monitoring test based on the piezoelectric active sensing and the wavelet packet analysis methods. Specimens are prepared to replicate in laboratory the composite rocks using cement. Two pairs of piezoelectric smart aggregates (SAs) are embedded in the composite specimens and an active sensing-based monitoring test is conducted during the shear test. Moreover, a wavelet packet analysis is carried out to compute the energy of the monitoring signal. The results demonstrate that signal amplitudes and peak values decrease significantly upon the shear failure of the composite specimens, whereas the shear failure and damage indices of the composite specimens increase abruptly.

Niu et al. [5] present a summary of existing measurement methods for tire-pavement friction estimation in airport runways. A multi-sensor information fusion scheme based on acoustic, optical and tread sensors, amongst others, is also proposed to estimate the friction coefficient and prospectively support aircraft maneuvering and decision-making processes in real-time.

Zhu et al. [6] propose an investigation into the improvement mechanism of steel slag on the low-temperature fracture behavior of permeable asphalt mixtures by acoustic emission (AE) testing. The authors use steel slag coarse aggregates to replace basalt coarse aggregates by volume at different levels (0%, 25%, 50%, 75% and 100%). The low-temperature splitting test with slow loading is used to obtain steady crack growth and the crack initiation and propagation of specimens are monitored by the AE technique in real time. Results show that the permeable asphalt mixtures with 100% steel slag have the optimal low-temperature cracking resistance. The analysis of the outcomes from the AE technique has proven that the incorporation of 100% steel slag reduces the shear events and restrains the growth of shear cracking in specimens at the macro-crack stage.

Kampczyk [7] presents a new approach to monitor the geometry of visibility triangles of railway level crossings (RLCs) using an electronic total station (TS) and a magnetic-measuring square (MMS). The angles of intersection between the road and the railway track axes and additional attributes related to the analysis and evaluation of general visibility conditions are accounted to demonstrate visibility. Results from a real case study prove the viability of the proposed approach and its potential impact on traffic safety at RLCs.

To achieve more accurate prediction of vehicle loads, Wu et al. [8] report a modified encoder-decoder architecture grafted with a signal-reconstruction layer to identify the properties of moving vehicles (i.e., velocity, wheelbase and axle weight) using merely the bridge dynamic response. A numerical simulation of a vehicle-bridge coupling model is used for this purpose. Outcomes prove that the method discussed in this study can predict traffic loadings, including properties of moving vehicles, without additional sensors or any vehicle weight label.

Lee et al. [9] present a smartphone-based dual-acquisition method system capable of acquiring images of road-surface anomalies and measuring the vehicle’ s three-axis acceleration upon their detection. Images are classified based on type and scale of anomalies and histograms of maximum variations of the acceleration in the gravitational direction are compared. Results demonstrate that the proposed approach can estimate accurately road-surface anomalies by linking them with a specific range of the maximum variation of acceleration.

Miwa [10] proposes a vibro–Doppler radar (VDR) measurement method to quantitatively evaluate rebar corrosion in reinforced concrete specimens. The author provides measurements of the rebar vibration ability forcibly vibrated in concrete by an excitation coil. It is demonstrated that the detection of the rebar vibration displacement by the proposed method is barely affected by moisture in concrete. Furthermore, an electrolytic corrosion test is carried out to monitor the displacement in the same specimens and results are compared to the rebar corrosion loss from VDR. The author proves that a relationship exists between the increase of the rebar vibration displacement process and the formation of corrosion cracks on the concrete surface.

Cao and Al-Qadi [11] present a numerical method to estimate asphalt pavement specific gravity from its dielectric properties. A three-phase numerical model considering aggregate, binder and air-void components is developed using an asphalt concrete mixture generation algorithm. The uneven air-void distribution in the three-phase model is generated through a take-and-add algorithm. The proposed model is capable of correlating pavement density and bulk and component dielectric properties. Additionally, the authors validate the model using field data collected with ground penetrating radar (GPR) and methods for calculation of the asphalt concrete mixture dielectric permittivity, including reflection amplitude (RA) and two-way travel time (TWTT) signal processing methods. Results indicate a higher sensitivity of the RA approach to surface thin layers compared to the TWTT method.

Gkyrtis et al. [12] report an integrated approach combining pavement sensing profile and deflectometric data that further evaluates indications of increased pavement roughness. Data including falling weight deflectometer (FWD) and road surface profiler (RSP) measurements are used in conjunction with additional geophysical inspection data from GPR. Based on the response from pavement modelling, promising results demonstrate that the method could proactively assists related agencies and asset owners in the health monitoring of transport infrastructure.

Fiorentini et al. [13] propose a methodology to correlate displacement measurements from interferometric synthetic aperture radar (InSAR) technique and measurements of road pavement surface roughness through laser profilometer along a 10 km-long pavement section. The authors use machine learning (ML) algorithms for prediction of average vertical displacements—detected by the persistent scatterer InSAR (PS-InSAR) technique—and the International Roughness Index (IRI) to quantify roughness. Research outcomes reveal a clear relationship between displacements from ML algorithms and the IRI values in several sections. The authors report that additional efforts are still required to reach a more comprehensive agreement between the two investigated methods. To achieve this goal, it is suggested to integrate traffic-related data, as well as structural and materials information into the ML algorithms.

Capozzoli et al. [14] investigate into the potentialities and limitations of the GPR and the electrical resistivity tomography (ERT) techniques with electrodes located both on the surface and in boreholes. An analogue model of a reinforced concrete frame is used for the experimental tests on full-size physical models. Results demonstrate the accuracy of GPR in reconstructing the rebar positions and detect potential defects, whereas the development of ad-hoc ERT methods are recommended to support the characterisation and monitoring of buried reinforced concrete foundation structures. A new structural monitoring approach based on the use of cross-hole ERTs (CHERTs) is also introduced to reduce uncertainties in data interpretation.

Research presented by Gagliardi et al. [15] aims to demonstrate the viability of using medium-resolution Copernicus ESA Sentinel-1A (C-band) SAR products and their contribution to improve current maintenance strategies in case of localized foundation settlements in airport runways. A geostatistical study is developed for the exploratory spatial data analysis and the interpolation between Sentinel-1A SAR data and topographic levelling data collected on a real-life airport runway, affected by known geotechnical settlements. The analysis provides ample information on the spatial continuity of the medium-resolution data in comparison with the high-resolution COSMO-SkyMed data and the ground-based topographic levelling data. Furthermore, a comparison between the persistent scatterers interferometry (PSI) outcomes from the Sentinel-1A SAR data—interpolated through ordinary Kriging—and the ground-truth topographic levelling information demonstrates the high accuracy of the Sentinel 1 data.

To conclude, the research presented in this Special Issue emphasizes the importance of using sensing systems for accurate and systematic monitoring and assessment of transport infrastructure and construction materials. Infrastructure is central in our society’s assets and commitment to ensure efficient management and maintenance should be continuous and reliable. It is observed that research has been developed to suggesting solutions for issues concerning both the short-term and the long-term performance of the infrastructure. To this effect, the application of emerging technologies for enhanced monitoring of infrastructure assets can potentially limit the risk of construction negligence, disruption and failure. Major efforts must be dedicated to find flexible solutions for infrastructure systems ageing with different time scales and requiring different repair and maintenance strategies. As well as experimental research conducted in laboratory, it is therefore important to demonstrate the effectiveness of sensing systems in the field. This could contribute to raise public awareness more effectively and facilitate a faster transition between research and industrial applications.
